# Supplementary material for: Transcriptional drifts associated with environmental changes in endothelial cells
Source: eLife. 2023 Mar 27;12:e81370. doi: 10.7554/eLife.81370 (PMC10168696; doi:10.7554/eLife.81370)
Supplement: Figure 2—figure supplement 2—source data 1. [file elife-81370-fig2-figsupp2-data1.zip › Figure_2-figure_supplement_2.html]

Profiler


Loading...

# How to use

- General
- Search
- Filter
- Combine
- Reset
- Similar
- Divergent
- Draw

To improve accessibility and analysis capabilities of the generated flow data, this website displays the data as a table, plots and with some analysis functionalities.
You can click on a gene in the table, to draw its plot to the right. The plot is drawn using, for each timestamp, the average value of available samples (the values shown in the table at columns 0h, 0.5h, 8h, 24h and 48h). Origin and slope values of this curve are also shown in the last columns of the table.
Below the plot, each sample data is displayed with another plot where each sample has its own curve (you can temporarily mask a curve on that plot by clicking on its name above the plot).

In the search bar you can type text and hit [ENTER] when you’re done. The text will be looked up in the table, using the gene symbol and description columns, and the first match will be selected. The search can take a few second, you can see a glowing circle on the left of the search field while it’s ongoing. If no hit is found in the table, the circle will be crossed out.

In the filter bar you can type text, and as soon as you type, the text will be looked up in the table, using the gene symbol, description, and module columns. Every line without a match will be masked, and you’ll see the filtered total entries number at the bottom of table reflecting the new displayed total.
You can also use the “Modules” dropdown list to filter on the selected modules (you can select multiple modules and you need to re-click on a selected module to deselect it).
You can combine both filter bar and modules dropdown list.

The combine tool allows displaying simultaneously multiple gene profiles in the same plot. Push the button to activate the “combine mode”. In that mode, each time you click on a gene in the table, that gene profile will be added to the plot. Re-click on a selected gene in the table to remove it from the plot. Push the “Combine” button again to leave the “combine mode”.
Note that when you use the search bar in combine mode, the first match will be automatically added to the plot.
Hitting the “Reset” button will deselect all genes in the table and clear the plot. You can also temporarily mask a curve on the plot by clicking on its name above the plot.

The “Reset” button will deselect all genes in the table and clear the plot.

This tool allows to find the most similar gene profiles compared to a selection of one or more profiles.
The similarity is defined by a value between 0 and 2, that you can select using the slider next to the “Similar” button. Then a range is defined for each available timestamp (the average value of the selection plus and minus the similarity value). When you hit the “Similar” button, all gene profiles in the table with all their timestamp in this range will be filtered in, and you will see the filtered total entries number at the bottom of table reflecting the new displayed total.
Note that if the plot displays multiple gene profiles (using the “Combine” button), they are all considered for the similarity computation (using average value of all displayed profile for each timestamp).

This tool allows to find the most divergent gene profiles compared to a selection of one or more profiles.
The divergence is defined by a value between 3 and 5, that you can select using the slider next to the “Divergent” button. Then a range is defined for each available timestamp (the average value of the selection plus and minus the similarity value). When you hit the “Divergent” button, all gene profiles in the table with all their timestamp outside this range will be filtered in, and you will see the filtered total entries number at the bottom of table reflecting the new displayed total.
Note that if the plot displays multiple gene profiles (using the “Combine” button), they are all considered for the divergence computation (using average value of all displayed profile for each timestamp).

You can click on the plot to draw one or multiple coordinates, generating a custom profile. That custom profile can be alone (if you drawn on an empty plot when no gene is selected in the table) or combine to one or more gene profiles (if they are selected in the table).
That custom profile is considered by the “Similar” and “Divergent” tools. It means that, for example, if you are looking for genes with a specific behaviour, you can just draw the desired curve and hit the “Similar” button.
Note that this custom curve can be partially drawn (missing some timestamp), those missing timestamp will just not be considered in the similarity/divergence computation.
Use the “Reset” button if you want to clear your custom curve.

##### Loading... Modules Combine Reset Loading... Similar Loading... Divergent

| symbol | gene name | ya93flow000 | ya94flow000 | ya95flow000 | ya96flow000 | ya93flow005 | ya94flow005 | ya95flow005 | ya96flow005 | ya93flow080 | ya94flow080 | ya95flow080 | ya93flow240 | ya94flow240 | ya95flow240 | ya96flow240 | ya93flow480 | ya94flow480 | ya95flow480 | ya96flow480 | 0h | 0.5h | 8h | 24h | 48h | module | slope | origin |
| --- | --- | --- | --- | --- | --- | --- | --- | --- | --- | --- | --- | --- | --- | --- | --- | --- | --- | --- | --- | --- | --- | --- | --- | --- | --- | --- | --- | --- |

##### Average Profile

##### Individual Replicates

| Key | Value |
| --- | --- |
| Key | Value |
| --- | --- |
