# Supplementary figures and images for: Transcriptional drifts associated with environmental changes in endothelial cells
Source: eLife. 2023 Mar 27;12:e81370. doi: 10.7554/eLife.81370 (PMC10168696; doi:10.7554/eLife.81370)

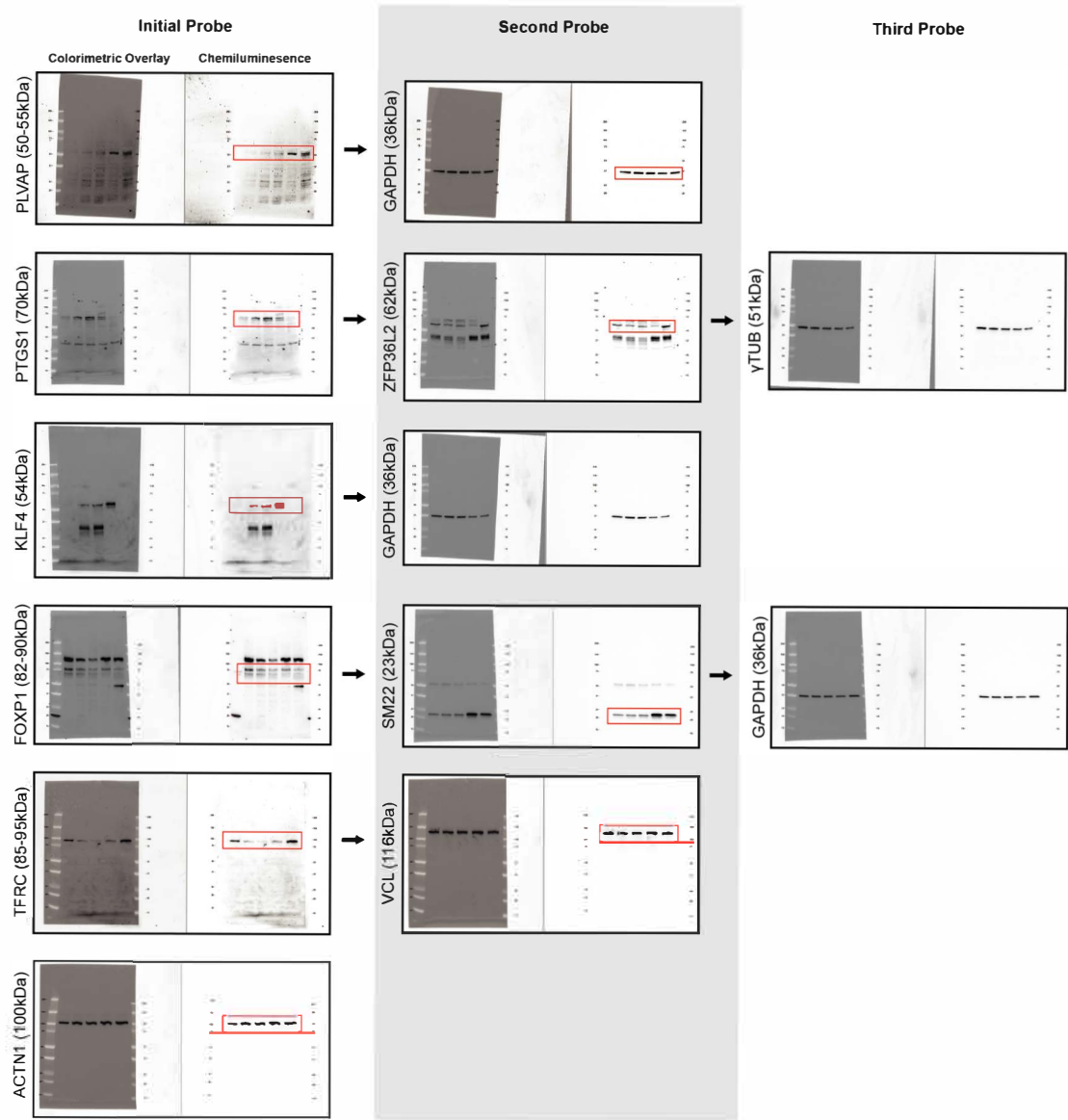

Supplement: Figure 4—source data 1. — On the left are six blots with the protein of interest and molecular weight of anticipated protein on the Y-axis with both colorimetric overlay and chemiluminescence image. The protein of interest is squared in red. If the blot had an additional second or third antibody probe it is depicted serially in a column with the protein of interest and molecular weight as marked. [file elife-81370-fig4-data1.zip › Figure 4source data 1.pdf]
