# Supplementary material for: Transcriptional drifts associated with environmental changes in endothelial cells
Source: eLife. 2023 Mar 27;12:e81370. doi: 10.7554/eLife.81370 (PMC10168696; doi:10.7554/eLife.81370)
Supplement: MDAR checklist [file elife-81370-mdarchecklist1.docx]

**Transcriptional drifts associated with environmental changes in endothelial cells**

Yalda Afshar, MD, PhD^1,2^, Feiyang Ma, PhD^2,3^, Austin Quach, PhD^3^, Anhyo Jeong^1^, Hannah Louise Sunshine^4,5^, Vanessa Freitas, PhD^6^, Yasaman Jami-Alahmadi, PhD^7^, Raphael Helaers^8^, Xinmin Li, PhD^9^, Matteo Pellegrini, PhD^2,3^, James Wohlschlegel, PhD^6^, Casey E. Romanoski, PhD^10^, Miikka Vikkula^8,11^ M. Luisa Iruela-Arispe, PhD^3,5, *^

^1^Division of Maternal Fetal Medicine, Department of Obstetrics and Gynecology, University of California, Los Angeles; ^2^Molecular Biology Institute, University of California, Los Angeles; ^3^Department of Molecular, Cell, and Developmental Biology, University of California, Los Angeles; ^4^Department of Molecular, Cellular & Integrative Physiology, University of California, Los Angeles; ^5^Department of Cell and Developmental Biology, Northwestern University Feinberg School of Medicine; Chicago, Illinois; ^6^Departament of Cell and Developmental Biology, Institute of Biomedical Science, University of Sao Paulo ^7^Department of Biological Chemistry, University of California, Los Angeles;^8^ Human Molecular Genetics, de Duve Institute, University of Louvain, Brussels, Belgium; ^9^Department of Pathology and Laboratory Medicine, University of California, Los Angeles; ^10^Department of Cellular and Molecular Medicine, University of Arizona, Tucson; ^11^WELBIO department, WEL Research Institute, avenue Pasteur, 6, 1300 Wavre, Belgium

*Corresponding author:

M. Luisa Iruela-Arispe, Ph.D.

Feinberg School of Medicine

Northwestern University

303 E. Superior Street – SQ 8-522

Chicago, IL 60611

[arispe@northwestern.edu](mailto:arispe@northwestern.edu)

Phone: 312-5037958

**Abstract:**

Environmental cues, such as physical forces and heterotypic cell interactions play a critical role in cell function, yet their collective contributions to transcriptional changes are unclear. Focusing on human endothelial cells, we performed broad individual sample analysis to identify transcriptional drifts associated with environmental changes that were independent of genetic background. Global gene expression profiling by RNAseq and protein expression by LC-MS directed proteomics distinguished endothelial cells *in vivo* from genetically matched culture (*in vitro*) samples. Over 43% of the transcriptome was significantly changed by the *in vitro* environment. Subjecting cultured cells to long-term shear stress significantly rescued the expression of approximately 17% of genes. Inclusion of heterotypic interactions by co-culture of endothelial cells with smooth muscle cells normalized approximately 9% of the original *in vivo*signature*.* We also identified novel flow dependent genes, as well as genes that necessitate heterotypic cell interactions to mimic the *in vivo* transcriptome. Our findings highlight specific genes and pathways that rely on contextual information for adequate expression from those that are agnostic of such environmental cues.

# **Introduction**

Endothelial cells define the functional integrity and response to hemodynamic blood forces on the luminal surface of blood vessels (1). They are also responsible for the selective trafficking of immune cells, regulation of metabolites and fluid extravasation to tissues (2-5). More recently, it has become clear that the endothelium provides instructive angiocrine signals required for the differentiation of tissues during development and for homeostasis of organs in the adult (6). In fact, it is challenging to identify a single pathological condition that could not be either worsened or improved by affecting the biology of blood vessels. Either through regulation of barrier function, anti-thrombotic properties, angiocrine or angiogenic capacity, endothelial cells have broad impact and therapeutic reach. Thus, there is a compelling incentive to define the mechanisms that control endothelial function and explore strategies to alter these functions as we work towards understanding disease etiology and processes leading to restore normal organ physiology.

Much of the knowledge accumulated on endothelial cell function has emerged through studies *in vitro*. The ability to grow endothelial cells under culture conditions has enabled investigators to identify growth factors that promote endothelial growth (7, 8), define the molecules involved in barrier function (9-11), and recognize discrete steps in leukocyte-endothelial selection and extravasation (12). However, a complete reductionist *in vitro* (culture) approach deprives endothelial cells from contextual information which could impact experimental read-outs.

As for all cells, the endothelial cell transcriptome is dependent on their native environmental milieu which includes homo- and heterotypic cell interactions, soluble factors, three-dimensional organization (13), and physical forces (14-17).This contextual information is removed when cells are placed *in vitro.* While endothelial identity and many biological aspects are retained, there is no frame of reference, meaning comparison to *in vivo* state, to determine what has been lost, and what could have been artificially gained, during the transition to an *in vitro* environment. Such gains and losses are likely to affect conclusions drawn from *in vitro* expression profiles. Yet, without an understanding of these changes, the validity of conclusions associated with experimental challenge remains uncertain.

To gain more clarity on the impact of culture conditions on endothelial cells, we set out to evaluate human umbilical vein endothelial cells directly upon removal from the cord (*in vivo* / cord) and after exposing the same cells to short and long-term *in vitro* culture. After defining the gene signatures changed in culture, we inquired as to whether *in vitro* environmental exposure to shear stress and interactions with smooth muscle cells were able to ameliorate the differential expression signatures and “correct” and “rescue” drifts. Through this process and relying on genetically identical *in vivo* transcriptome, we identified groups of genes exquisitely dependent on long-term shear stress and others dependent on heterotypic cell interactions. Importantly, we also identified a large cohort of genes that were unable to regain levels comparable to *in vivo* settings and others that were artificially induced by exposure to culture conditions. Together, this work has implications for enabling investigations of endothelial cells with improved fidelity to *in vivo* phenotypes that should improve reproducibility and translation of experimental findings.

# **Results**

*Transcriptional drifts associated with the transition of in vivo (cord) to in vitro (culture)*

To uncover changes on endothelial cells as result of exposure to culture conditions, we evaluated the transcriptome of endothelial cells isolated from human umbilical cord veins. Half of each participant’s cell preparation was freshly processed for RNA isolation (referred to as “cord” or “*in vivo*”) while the other half was placed under culture conditions (referred to as “culture” or “*in vitro*”). Cells were subsequently passaged and evaluated at “early” passage (P 2-3) and “late” passage (P 7-8) to capture transcriptional differences between cellular environments that were common amongst all seven patients regardless of fetal sex or genetic background (Figure 1A, Supplementary File 1). Patient demographics with paired maternal-fetal outcomes were provided in Table 1, and each patient had matched cord, early passage, and late passage paradigm. Principal component analysis (PCA) of bulk RNA sequencing (RNAseq) transcriptional profiles revealed that cord versus culture/*in vitro* environments were the dominant factor influencing measured expression levels (Figure 1B-C, Supplementary File 1). PC1 captured 47.4% of the total variance whereas PC2 only accounts for 11.1%. PC2 appeared to represent the differences between early and late passage but these conditions did not segregate from each other as clearly as cord versus *in vitro* culture. As such, all comparisons to culture conditions are subsequently made with early (*in vitro)* cells in culture.

Approximately half of the expressed genes were differentially expressed between cord and culture conditions (4,532-4,645 genes overlapping), whereas the transcriptomic signature was very similar between early and late culture (11,706 genes overlapping) (Figure 1D). As such, we considered only differences between cord and culture signatures going forward (Figure 1E-G, Supplementary File 1, Figure 1-figure supplement 1-2).

Genes with robust changes in expression are highlighted in Figure 1E-F. Amongst several signatures, we observed that TGF-beta and BMP target genes were reduced under culture. Some of the most *in vivo*-specific transcripts were related to the extracellular matrix; while several genes specific to the *in vitro* environment associated with the cell cycle (Supplementary File 1). We also found that the most highly expressed genes across patients and environments demonstrated minimal variation across individuals and considerable variation between environments (Figure 1F, Supplementary File 1). As expected, we found that endothelial cells lose expression of flow-responsive genes (*KLF4, KLF2*) once placed under culture conditions, whereas they quickly acquire proliferation-related genes (*CCNB2, CCNA2, CDCA2*). Perhaps more surprising was that transition into culture promotes a significant decrease in transcripts associated with extracellular matrix genes (*COL23A1, MMP28, FBLN2, ELN, COL1A2, COL6A3*), cytokine (*CXCL2, SOCS3*, *TGFB3, CTGF*), and early response genes (*FOS, ZFP36, JUNB*) (Supplementary File 1, Supplementary File 4). In addition to increased expression of cell cycle genes in culture, transcripts associated with survival and a pro-angiogenic phenotype were also upregulated (e.g., *APLN, BAX, CCN*, *CCNB2, CCNBA1, CEPH1, CDCA7l, CDCA2, MDM2*). Further, the significant increase of VEPH1 under culture conditions was of particular interest as the protein product of this gene is associated with suppression of TGFß1, FOXO and Wnt signaling (18).

Gene Ontology term enrichment of differentially expressed genes was performed using GO biological processes in order of significance. Significant terms, defined using hypergeometric p-values and enrichment factors, were hierarchically clustered based on similarities among gene members into networks (Figure 1G).

In the network, terms are represented by a node with its size proportional to the number of differentially expressed genes in that term. Focusing on genes expressed uniquely in cord relative to culture, we found enrichment of transcripts with documented involvement into blood vessel development, skeletal system development (mostly the TGFß family), heart / blood vessel development, ossification (extracellular matrix genes), and cytokine production (Figure 1G).

To determine whether the identified changes were supported by similar drifts at the protein level, validation of the transcriptomic signature was performed by comparing cord and *in vitro* protein extracts by untargeted LC-MS based proteomics. PCA analysis of relative protein abundances was conducted for seven matched individuals from the cord and early culture. The analysis demonstrated clear separation of the experimental conditions (Figure 1H). In agreement to the RNA level differences, there were significant changes in protein expression between the environments. Albeit not as remarkably different than the transcriptomic read-outs (likely due to depth of coverage and statistical power), we identified an -omics signature of proteins specific to cord (about 160/3000 proteins) and to early culture (about 411/3000 proteins) (Figure 1-figure supplement 2E). These differences are presented in Figure 1-figure supplement 2F.

To explore the degree of overlap between RNA and protein, we compared the results of differential transcripts using the cord *versus* culture analysis to that of protein cord *versus* culture analysis (Figure 1-figure supplement 2G). The relationships between t-statistics between cord and culture across -omic layers revealed significant correlation between RNA and protein signatures (r=0.4, p=1x10^-07^) (Figure 1I). Consistent with prior findings, the data revealed low expression of cell cycle proteins and high expression of flow-responsive proteins in the cord (ex-vivo) proteomics profile (Figure 1I, Supplementary File 2, 9).

*Global transcriptional changes affected by shear stress*

A large number of genes associated with transition from cord to culture appeared to be flow-related, we explored the potential to ameliorate these differences by imposing shear stress on cultured cells. This approach is warranted by observations that once placed under laminar flow, endothelial cells significantly change their morphology and reduce proliferation resembling in vivo conditions (19). Further, the onset of flow is associated with significant transcriptional increase in flow responsive genes, like *KLF2* (20-22), which is one of the cord-specific transcripts (Figures 1,2, Supplementary File 3). We thus performed two comparisons: 1) static culture to flow cultures (Figure 2A), and, 2) each to cord endothelial cells (Figures 2,3, Supplementary File 3).

Indeed, we found that shear stress significantly rescued the expression of approximately 17% of genes including targets of BMP and Notch signaling known to be sensitive to flow. At the transcriptional level, the effect of physical forces, particularly laminar, oscillatory and disturbed flow has been extensively investigated (23-26). These investigations have been instrumental to clarify the effect of shear stress on endothelial cells. Nonetheless, previously published studies focused in evaluating flow responses at short and long time points in relation to static cultures. We took advantage of having isolated RNA directly from cords, allowing for comparisons between *in vivo* and *in vitro* (static and flow) conditions using genetically identical backgrounds.

First, principal component analysis of matched patients (n=4, Table 1) demonstrated static cells *in-vitro* and under 30-minutes of flow displayed relatively similar global transcriptional signatures. Differences were apparent on PC1 with flow, defined as 8-48 hours of laminar shear stress exposure (Figure 2B). Figure 2C provides a clear delineation and transcriptomic signature as a function of static (control and 30min) versus longer time points (8, 24, 48hr of flow). Significant changes (log10FC) were noted between static and flow cultures (Figure 2D and Supplementary File 3-4), with *IGFBP5, ELN, KLF4, ETPR1*, and *TGFBR3* significantly dependent on flow for their transcriptional increase. Correlation scatter plots of the cord versus culture (x-axis) were compared to time under flow (y-axis) and this analysis showed a time-dependent positive correlation to the cord transcriptome (vs culture) (Figure 2E). Progressive time under flow from up to 48-hours of shear stress (flow) revealed that the transcriptional signature of cells correlates more specifically to that of the cord than with static cultures. Initially, the correlation coefficient was insignificant (r=-0.035, p=0.004) with progressive changes to the point that by 48-hours of shear stress the correlational coefficient to cord reached r=0.34, =-8.0x10^-9^, which is significantly different than static culture (Figure 2E). Collectively these data offer proof that drifts in the transcriptome of endothelial cells under culture can be partially rescued by exposure to laminar shear stress. To improve the data accessibility and analysis capabilities of the generated transcriptomic flow data, we implemented an open-source website, *Flow Profiler,* to display the data as a table, plots and with some analysis functionalities (Figure 2-figure supplement 2; Figure 2-figure supplement 3).

A marked change towards the cord state was noted also by pathway analysis. Specifically GO terms associated with blood vessel development (*EDN1, BMP2, BMP4, TGFPR3, ITG1BP1, HES1, HEY1*), regulation of cellular protein location (*ITGA3, RACK1, PTPN9, SPTBN1*), and cellular response to laminar fluid shear stress (*ASS1, KLF2, KLF4, MAPK7, NFE2L2*) were regained by long-term exposure to flow (Figure 2F). Gene set enrichment analysis of differential expressed genes in cultured endothelial cells under flow (versus static) revealed gene annotations related to an acute inflammatory response, heart morphogenesis, second messenger-mediated signaling, and ossification (related to BMP and TGFbeta responses). We also found tRNA and rRNA metabolic processes were silenced under flow (Supplementary File 3-4, Figure 2-figure supplement 1, Figure 3-figure supplement 1).

*Imposing shear stress in vitro partially rescues the in-vivo signature*

Superimposing the cord and culture signatures (from Figure 1) with the static versus flow experiments (from Figure 2) clarifies how the transcriptional profile of cultured cells under flow approximates the *in vivo* transcriptome better when compared to static states (Figure 3A, across PC1, Supplementary File 3-4). PC1 primarily displays the differences between Cord and Culture samples (we interpret the PC2 to represent differences between short-term and extended flow). Since the extended flow samples are in the middle position between cord and late culture, we interpret this as a partial rescue of the differences imparted by culture. This shift was also noted by evaluating total number of transcriptional changes up or downregulated (Figure 3B). In fact, much of the cord signature overlapped with genes that were rescued or attenuated under flow and paralleled those expressed by the cord. Specifically, the incorporation of shear stress to the *in vitro* static conditions attenuated the variability between cord and culture with a drift recovery in 17% of the genes (Figure 3).

To identify cohorts of genes altered by shear stress and that approximate the *in vivo* environment (“rescued”), we performed a transcriptome-wide weighted gene co-expression network analysis (WGCNA). This approach led us to identify 36 co-expression modules, revealing gene groups that are co-enriched in either cord or culture environments, or in static versus flow conditions (Figure 3C, red: down; blue: up, Supplementary File 3-4, Figure 3-figure supplement 1). Relative expression values for the most expressed modules across each of the patients are illustrated in Figure 3-figure supplement 1D-E and then culminated in summary in Figure 3D.

Superimposing the cord transcriptome on the flow transcriptome, highlighted co-expressed modules with significant enriched directionality (concordance of upregulation or downregulation) in cord and culture transcriptomes (Figure 3C). Specifically, we compared the differential gene expression of the cord to that of the cultured cells under flow. Using the differential gene expression, gene ontology (GO) network analysis of WGCNA demonstrated that differential modules were selectively increased (blue) and decreased (brown) by long-term exposure to shear stress (Figures 3E-H). The blue module (2,185 genes, r=0.71, p=3e-04) was increased in the cord and under flow conditions as compared to static culture. The blue module showed increased transcriptional concurrence with the cord and this was progressive with time under flow (r=0.8, p=4e-04). Although exposure to shear stress partially recapitulated the cord environment (Figure 3E), this was not the case for all the transcripts, highlighting signatures that are exquisitely flow-dependent and others that are flow-independent and likely regulated by alternative factors, such as heterotypic cell interactions or in vivo metabolites. Notably, the genes and GOs associated with this module included blood vessel development and leukocyte activation (Figure 3F). The brown module was decreased both in the cord and in flow as compared to static cells (1,408 genes, r=-0.9, p=3e-08). The brown module was defined by cell cycle and cell cycle checkpoints, was less expressed in cord (vs culture) and in flow (vs static, r=-0.62, p=0.01). These genes gained expression in culture, yet flow reverted their phenotype to lower expression, as was evident in cord (Figure 3G-H, Table 2, Supplementary File 5). In summary, this network analysis uncovered co-expressed gene signatures that are sensitive to shear stress (induced, aka blue module and repressed, aka brown module) and represented *in vivo*.

In addition, the robust dataset identified transcripts previously unknown to be altered by laminar shear stress (Supplementary File 3). To confirm the reproducibility of a few transcripts at the protein level, we validated by Western blot three examples found to be upregulated, downregulated or unchanged that were further in relation to their levels *in vivo* (endothelial lysates from cord umbilical vein) (Figure 4, Figure 4-figure source data 1). Detailed evaluation of the time kinetics for transcripts uncovered important nuances, for example, the extended time course (48hrs) of shear stress is important for some transcripts. For example, thioredoxin-interacting protein (TXNIP), a stress-responsive protein that inhibits thioredoxin and previously thought to be reduced by exposure to 24h of shear stress [PMID:15696199], exhibits a drastic increased by 48h of laminar flow. A list of the top 30 transcripts that are altered from in vivo (cord) to in vitro is shown in Table 2. Table 3 shows the extent of concordant and discordant rescue by exposing cells to laminar shear stress. In addition, Figure 2-figure supplement 2 introduces a platform “Flow Profiler” to interrogate the behavior of any gene under flow.

*Co-culture with smooth muscle cells further rescues the in-vivo transcriptional profile of endothelial cells*

Given these global differences between cord and culture, we asked whether the differential gene expression was also affected by heterotypic cell interactions, namely with smooth muscle cells. To address this question, we leveraged single cell RNA sequencing (scRNAseq) technology to obtain transcriptomes of individual cells isolated from endothelial cells in a homogenous culture (mono-culture, MC) versus endothelial cells co-cultured with smooth muscle cells (co-culture, CC). The approach was aimed at further approximating contextual environment and obtain signatures responsive to those changes (Figure 5A). We profiled technical replicates of primary endothelial cells, primary smooth muscle cells (individual mono-cultures), and co-cultured endothelial cells and smooth muscle cells all plated to confluency using scRNAseq (Supplementary File 6, Figure 5-figure supplement 1). Endothelial and smooth muscle cells were isolated from the same cord eliminating potential confounding factors associated with genetic variations. In total, 51,000 cells were sequenced with an average of 3,402 genes and 18,740 transcripts per cell. Individual samples were independently analyzed to confirm correlation between triplicates, normalized and then combined for analysis. Unsupervised clustering demonstrated the cells cluster by origin (Figure 5B-E). We then confirmed cell clusters as endothelial cells (*PECAM1* and *CDH5*, Figure 5C, E) and smooth muscle cells (*ACTA2* and *TAGLN*) (Figure 5-figure supplement 1C).

Transcriptomic profiles that defined each cluster was performed by Seurat and this information offered initial insight on transcriptional shifts that occurred consequently to heterotypic cell interactions. As shown by heatmap (Figure 5F), clear differences were noted when endothelial cells were in mono-culture (MC) versus co-culture (CC). Specifically, co-culture prompted a reduction in NOTCH target genes (*FABP4, GJA4, FABP5, HEY*) and a clear induction in TGFß downstream targets (*SERPINE1, IGFBP7, SOX4, TIMP1*) (Figure 5F). Ingenuity pathway analysis provided further clarification as to the functional impact related to presence of smooth muscle cells. As shown in Figure 5G, the major signaling pathways and transcriptional regulators that prompted transcriptional drifts on endothelial cells by co-culture included TGFß, VEGF, TP53, HTT, MYC, TNF, EDN1, SP1 and HGF. We calculated a module score using the expression of downstream targets for TFGß1 and VEGFA identified by ingenuity pathway analysis and found a significant increase upon co-culture for both (Figure 5H). This is entirely surprising as smooth muscle cells provide a source for these two cytokines. Activation of the TGF-beta pathway results in shifts in extracellular matrix proteins, MMPs, and integrins (Figure 5I) and it is further supported by transcriptional increases in TGFß receptors ACVRL1 and ENG. Interestingly, co-culture conditions resulted in an increase of clathrin-related genes (*AAK1, AP2B1* and *CLTB*) and a decrease in caveolin-related genes (*CAV1* and *CAV2*) (Figure 5J). These changes occurred with no significant alterations in *CDH5, ERG, NOTCH1* and *JAG1* (Figure 5K).

Naturally the next question focused on which signatures impacted by heterotypic cell interactions yield a rescue of the *in vivo* condition. To delineate these transcriptional relationships, we overlapped scRNA sequencing data obtained from cord-derived endothelial cells and compared them to the mono and co-culture endothelial transcriptomes (Figure 6A-C, Supplementary File 7-8). Interestingly, global transcriptional profiling in UMAP showed a shift of co-culture towards cord (Figure 6A). In-depth analyses of the data using Seurat, GOs and ingenuity pathways revealed cohorts of genes that were indeed rescued (either up or downregulated) and genes that were not rescued by the co-culture condition. Examples of those categories are shown in Figure 6D and group analysis by dot blot as displayed in Figure 6E. Genes rescued by co-culture relate to NOTCH signaling (*HES1, FABP4*) and TGFß (*ENG*). In addition, we found that clathrin pathways, noted to be increased by SMC-co-culture (Figure 5) were indeed part of the *in vivo* signature displayed by endothelial cells in the cord (Figure 6F) with upregulation of transcripts for *AAK1* and *EPN2*. Co-culture also was responsible for rescue of TJP1, responsible for tight junctions and two transmembrane proteins that regulate calcium homeostasis (TMEM165 and 203) (Figure 6F). Interestingly we noticed a decrease in *IRF7* and *VASH1* under co-culture that also approximated the cord condition. In summary, co-culture of endothelial cells with smooth muscle cells normalized networks related to cell growth and differentiation, clathrin-vesicle related genes, and recovered targets downstream TGF-beta, recovering approximately 9% of the original cord (*in vivo*) signature (Figure 7)*.*

**Discussion**

Endothelial cells are characterized by a unique set of genes collectively referred to identity genes (i.e. *CDH5, PECAM1, ERG*) and a group of genes whose expression level varies according to stressors and environmental conditions. Precise information of both groups holds relevance to the interpretation of findings related to any experimental challenge. Despite the broad utilization of cultured endothelial cells, drifts in the transcriptional profiles upon expansion *in vitro* have not been rigorously addressed. Here, we undertook parallel transcriptomic analyses using genetically identical matches to determine the impact of the environment on cell culture and define whether specific signatures could be regained by changing environmental settings that will best approximate the native biological state.

To minimize confounding factors related to intrinsic genetic differences, we performed parallel transcriptomic profiling. Seven pairs of freshly isolated *versus* cultured endothelial cells were used for the initial profiles and the findings from these were validated against proteomics from seven independent pairs. Four additional cohorts were used to compare static *versus* flow *versus* freshly isolated conditions and single-cell RNAseq was subsequently used in the co-culture experiments. Our findings highlighted signatures that were uniquely associated with long-term exposure to shear stress *in-vitro* that parallel expression profiles *in-vivo*. We also identified signatures dependent on heterotypic, endothelial-smooth muscle cell interactions that were lost *in-vitro*, but a hallmark of the *in-vivo* state. The findings offer an important resource to query how expression profiles of specific genes change in relation to a subset of environmental conditions.

A major adaptation that cells must acquire when placed in culture relates to cell proliferation. Once seeded, endothelial cells undergo significant expansion that is thought to be attenuated or suppressed at confluency. Nonetheless, we demonstrate that high levels of transcripts related to cell cycle, mitosis, and DNA repair mechanisms are still present at confluency and represent the single most significant alteration when comparing freshly isolated cells to genetically identical cohorts *in vitro*. Similarly, there are significant alterations in cytoskeletal dynamics and focal adhesions that are artificially elevated *in vitro*, compared to *ex-vivo*.

Recapitulating the native flow seen by endothelial cells by exposure of static cultures to shear stress resulted in a significant shift towards ex vivo (freshly isolated cells) signature. Much has been done to understand transcriptional responses to flow. Most of these have been focused on early responses in the absence of *in vivo* genetically matched counterparts (27-32). Previous studies have described the effects of cell culture on endothelial cells with changes in gene expression (33-36) or characterized the change in differential gene expression with shear stress (37-39). The novelty of our study is the systematic analysis of “recovery” and “not recovery” based on changes in shear stress and exposure to smooth muscle cells.

Our data found agreement with previous findings of short time exposure to shear stress we noted an impressive induction of KLF2 and KLF4 (40). However, longer exposure to laminar flow (8, 24, 48hrs) progressively increased the resemblance to the *in vivo* transcriptome, as noted by correlation coefficients. Specifically, we found that two major pathways and their downstream genes were regained by long-term flow: BMP and NOTCH signaling. Importantly, it has been recently shown that BMP signaling is significantly potentiated by flow (41). Indeed, several SMAD targets were rescued by incorporating long-term flow into cultures. Similarly, NOTCH target genes (*HES, HEY*) regained levels similar to those captured in freshly isolated preparations. These findings are congruent with recent studies demonstrating that NOTCH signaling was increased by flow and mechanosensing (42). An unexpected gene ontology signature regained by shear stress included proteins associated with cellular localization, such as ITPR1, IGFBP5, DLL1, among others, highlighting the role of laminar shear stress in endothelial cell polarity. Not surprisingly, the most significant protein changes coincide with significant corresponding changes in RNA but the most significant changes in RNA did not coincide with significant changes in corresponding protein levels (Supplementary File 9).

Alterations in junctional proteins and cytoskeletal architecture were recovered in endothelial-smooth muscle cell co-cultures. Co-culture of endothelial with smooth muscle cells also induced TGF-ß downstream targets in the endothelium, including several extracellular matrix proteins and integrins which brought further alignment to the *in vivo* transcriptome. In addition, smooth muscle cells significantly reduced the prominent proliferative signature of endothelial cells and promoted a partial recovery in endothelial cell differentiation. Specifically, this included ENG and integrins regulated by TGFB1 (ITGB1, ITGA1, ITGA5), as well as several extracellular membrane proteins (COL1A1, FN1, TIMP1, SERPINE1) **(43-45)**. It can be postulated that loss of architecture *in vitro* could induce the loss of expression of acute phase transcripts, as seen with injury of the aorta *in vivo* (46). These endothelial-heterotypic crosstalk have been shown essential during development and altered in vascular pathologies such as aneurysms (47).

Exposing endothelial cells to culture conditions does not appear to significantly affect cellular identity. Transcriptional levels of CDH5, PECAM1, ERG, Claudins, Sox(s), and other so-called endothelial markers were not significantly impacted. ERG is essential for regulation of CDH5, VWF, and NOS3 as well as a hallmark of endothelial cell lineage (48-52).

Despite notable strengths, our study has several limitations, including the individual participant heterogeneity which introduces inter-subject variability and lack of functional read-outs of the biology described. We acknowledge this limitation, albeit patient diversity provides a more representative and realistic model to understand biology and disease. It should be also stressed that the present study focuses on HUVECs and does not delve into the remarkable heterogeneity of organ-specific vascular beds that might respond differently to shear stress. Additionally, we and others (53) have noticed distinct populations in the PCA of cultured ECs during our single-cell RNAseq studies that were not explored here. Evaluation as to these subpopulations, which are also noted in the aorta in vivo (53) reflect transcriptionally distinct groups or different states of cyclic expression patterns and requires a more thorough analysis and lineage tracing studies that are distinct from the objective of the question posed here. This second type of analysis, along with assessment of chromatin states (ATACseq) may provide clear-cut cell-subtype and state specific information. Finally, considerations of how *in vivo* metabolites influence the transcriptional read-out of the endothelium were not explored here. It is likely that metabolites may aid in further correcting shifts from in vivo to in vitro conditions that were not affected by the two factors evaluated here. We found that 26% out the 43% of transcriptional alterations could not be recovered by either shear stress (which rescued 17% of the changes) or by contact with smooth muscle cells (that rescued 9% of the changes). There are still 17% of transcriptional drifts that could not be recovered.

The ability to grow and study endothelial cells *in vitro* has enabled investigators to ask questions under well controlled, yet artificial, conditions. The consequences associated with phenotypic alterations of ex vivo expanded cells remain unknown despite ample evidence that culture conditions exert profound influence upon cellular biological properties (54-57). We defined a transcriptionally unique fingerprint of endothelial cells immediately removed from the cord and mapped how environmental changes uniquely impact this profile. These -omics analyses offer information that can guide researchers to have a better understanding of intrinsic mechanisms that are not captured when studying signaling pathways and molecular processes in culture. Appreciating these nuances and recapitulating intrinsic shear stress and heterotypic cell interactions will help propel reprogramming efforts for the generation of a more representative *in vivo* model system allowing investigators to better interpret genetic modifiers that affect or are affected by endothelial cells.

**Methods**

*Endothelial cell isolation, culture and RNA extraction:*

Human umbilical cords were collected under Institutional Review Board (UCLA IRB#16-001694) at time of birth. Umbilical cords were processed within 2-4 hours from time of birth and cells were isolated as previously described (58). All samples were collected from participants who provided signed written informed consent and were de-identified immediately after cords were obtained. Table 1 describes the clinical details of the participants/donors. The umbilical cord was clamped bilaterally, cut at least into two fragments, and placed in HBSS at room temperature. The umbilical vein was cannulated with a 18G animal feeding needle with a blunt tip in the direction of oxygenated blood flow from the placenta to the fetus. Subsequently, the umbilical vein was serially washed with 20 mL of HBSS (HBBS) 3x to remove blood cells from the lumen. For collection of *in vivo* samples: 1mL of RLT from RNeasy Micro Kit (Qiagen, Germantown, MD) flushed through closed circuit and re-aspirated with the distal end of the umbilical cord clamped and stored in -80-degrees C until all RNA was ready to be extraction. The length of time to obtained cells was approximately 30-60 minutes from cord clamping at delivery/birth.

For isolation of endothelial cells for culture, the other half of the umbilical cord was flushed with 8 mL of collagenase-2 (210 IU, Worthington Biochemical, Lakewood, NJ) and the exudate was further incubated at 37^o^ C for 20-minutes to dissociate cells. Collagenase was inactivated with the addition of equivalent volume 10% fetal bovine serum (FBS) with MCDB 131 media (VEC Technologies, Rensselaer, NY). The cells were pelleted, resuspended in media, plated and cultured for 30min on tissue culture treated dishes in humidified incubator at 37°C and 5% CO_2_. After the short incubation period (30min), the plates were washed to remove non-adherent cells. After cells became confluent, additional purification steps were conducted (Miltenyi Biotec #130-091-935) and the purity of the endothelial isolates was evaluated by FACs analysis, immunocytochemistry and, in the case of the in vivo counter parts (RNA from the umbilical cords) we also used scRNAseq. The information on the purity of the isolated cells is shown in Figure 1-figure supplement 1. For culture cells, they were passaged using 1x Trypsin (Fischer Scientific, Waltham, MA) and collected with RLT for RNA extraction at early passage (passage 2-3) and at late passage (passage 7-8). Subsequently all RNA was extracted in tandem using RNeasy Micro Kit (Qiagen, Germantown, MD). Contamination with genomic DNA was eliminated with incubation of DNase I at room temperature. Agilent Bioanalyzer 2100 system (Agilent Technologies, Santa Clara, CA) was used to assess RNA integrity and Qubit (Invitrogen, Carlsbad, CA) for RNA concentration and purity.

For co-culture experiments, primary umbilical smooth muscle cells were isolated from the same genetically identical cords after removal of the endothelium and expanded in vitro for two passages. The purity of the smooth muscle cells was tested by scRNAseq (Figure 1-figure supplement 1). Smooth muscle cells were then seeded at 50% confluency onto the culture plate 24h prior to adding HUVECs to reach a confluent density. After an additional 24h co-cultured cells were trypsinized for scRNAseq experiments.

*Sequencing data samples and mapping:*

Library preparation was performed using TruSeq Total RNASeq Kit (Illumina, San Diego, CA) according to the manufacturer’s instruction. Sequencing was conducted on an Illumina HiSeq 4000 (RNAseq) and NovaSeq S2 (scRNAseq) instrument (Illumina, San Diego, CA) at the University California Los Angeles. Sequencing parameters were optimized for 50 bp single-end reads at a depth of 30,000 million reads/sample. Reads were mapped to the hg38 build of the human genome with Bowtie2 (59) and RNAseq reads were mapped with STAR (60). RNAseq experiments that measured accessibility and expression in different environment (cord versus culture) were all conducted at least twice. Benjamini-Hochberg false discovery rate (FDR) method was used to correct for all multiple testing in this study with a significance threshold of FDR <0.05. No explicit power analysis was used to compute sample size.

*RNAseq gene expression analysis:*

Gene expression analysis was conducted using R software. First, each raw count TPM gene expression profile was log10 transformed and rescaled to zero-mean and unit-resolution for both the cord vs culture and flow vs static datasets. Data was adjusted for batch effects using an empirical Bayes framework with the ComBat function from the sva package; no covariates were included in the model and the algorithm was set to use non-parametric adjustments. The expression of individuals genes was screened for associations with experimental treatments using biweight midcorrelation, a robust correlation measure, with the bicorAndPvalue function from the WGCNA package. Individual genes were also tested for associations with experimental treatments using Welch’s t-test using the base R t-test function, adopting a Bonferroni-corrected significance threshold (p < 2.5e-6). Principal components analysis (PCA) was conducted following gene-wise scaling to zero-mean and unit-variance. Weighted gene co-expression network analysis was conducted using the blockwiseModulesvfunction from the WGCNA package; the network soft-thresholding power was set to 3, the network type was set to “signed hybrid”; and the entire gene set was used for module detection by adjusting the maxBlockSize. The data can be found on the Gene Expression Omnibus (GEO) under the GEO accession number GSE158081. Both STRINGv10(61) and Metascape(62) was used to generate differential gene expression figures.

*Flow Profiler: transcriptomic web application*

The web application for viewing flow transciptomic data was built to display data as a table or plot and allows for easy accessibility to all investigators. The application was developed using html, JavaScript and CSS. A plot is drawn for each gene, at the indicated times and the average value of all available samples is displayed. A searchable/filtrable table gather the average values per gene plus the origin and slope of the resulting curve. Origin and slope were computed using the linear regression functions from Excel 2019 (INTERCEPT and SLOPE). The website provides a set of functionalities supporting the analysis of gene profiles. In addition to basic tools allowing search, filter, and combination of multiple profiles on one plot, two tools allow to find the most similar or divergent profiles compared to a selection of one or more profiles. The similarity is defined by a value between 0 and 2. Then a range is defined for each available timepoint (the average value of the selected gene plus and minus the similarity value). All profiles with all of their timespoints within this range are filtered. Divergence works in a comparable way, but only profiles with an average value outside the defined range are filtered. The two functions can also be used with an artificial curve as the comparison basis (the user can draw the desired curve on the plot).

*LC-MS based proteomics:*

Protein samples were reduced and alkylated using 5mM Tris (2-carboxyethyl) phosphine and 10mM iodoacetamide, respectively and digested by the sequential addition of trypsin and lys-C proteases, as described (63, 64). The digested peptides were desalted using Pierce C18 tips (Thermo Fisher Scientific, Waltham, MA), dried and resuspended in 5% formic acid, and fractionated online using a 25cm long, 75 uM inner diameter fused silica capillary packed in-house with bulk C18 reversed phase resin (1.9 uM, 100A pores, Dr. Maisch GmbH). The 140-minute water-acetonitrile gradient was delivered using a Dionex Ultimate 3000 UHPLC system (Thermo Fisher Scientific, Waltham, MA) at a flow rate of 300 nl/min (Buffer A: water with 3% DMSO and 0.1% formic acid and Buffer B: acetonitrile with 3% DMSO and 0.1% formic acid). Peptides were ionized by the application of a distal 2.2kv and introduced into the Orbitrap Fusion Lumos mass spectrometer (Thermo Fisher Scientific, Waltham, MA) and analyzed by tandem mass spectrometry (MS/MS). Data was acquired using a Data-Dependent Acquisition (DDA) method comprised of a full MS1 scan (Resolution = 120,000) followed by sequential MS2 scans (Resolution = 15,000) to utilize the remainder of the 3 second cycle time. The mass spectrometry proteomics data have been deposited to the ProteomeXchange Consortium via the PRIDE (65) partner repository with the dataset identifier PXD020958 and 10.6019/PXD020958. Data analysis was performed using the MSGF+ search engine (66) via the target-decoy strategy against the EMBL Human reference proteome (UP000005640 9606). The identification false detection rates (FDRs) at the peptide-spectrum-match (PSM) were defined using Percolator, protein identification confidence was estimated via the stand-alone implementation of FIDO such that analytes had respective q-values at or below 0.01 at both PSM and protein level (67-69). Extracted ion chromatograms were calculated for each peptide using Skyline (70). The MSStats R-package was used to normalize across runs using quantile normalization, summarize peptide-level intensities into a protein-level abundance, and perform statistical testing to compare protein abundance across conditions (71).

*Shear stress application:*

Confluent endothelial monolayers were grown on tissue culture treated 6-well plates (Falcon #08-772-1B) in complete MCDB-131 media (VEC Technologies # MCDB131-WOFBS) plus 10% FBS (Omega Scientific #FB-11) containing 4% dextran (Sigma-Aldrich #31392) for approximately 12-18 hours and then subjected to shear stress (130rpm) in new medium containing 4% dextran (Sigma-Aldrich #31392) for indicated time intervals and cultured alongside static controls. Orbital shear stress (130 rpm) was applied to confluent cell cultures by using an orbital shaker positioned inside the incubator as previously discussed (72). The shear stress within the cell culture well corresponds to arterial magnitudes (11.5 dynes/cm^2^) of shear stress. To reduce issues associated with uniformity of shear stress, the endothelial cell monolayers in 6-well plates were lysed after removing center region using cell scraper (BD Falcon #35-3085) and washing with 1X HBSS (Corning #21-022-CV). A 1.8cm blade was used circumferentially to remove the center of the monolayer that did not see the higher shear stress.

*Single-cell sequencing and data analysis:*

Single cells were isolated from umbilical cord flushes as described above. To keep the processing time between tissue harvesting and single-cell lysis at a minimum, no further cell type enrichment step was performed. For the generation of single-cell gel beads in emulsion, cells were loaded on a Chromium single cell instrument (10x Genomics, Pleasanton, CA) with an estimated targeted cell recovery of ~5,000 cells as per manufacturer’s protocol. In brief, single-cell suspension of cells in 0.4% BSA-PBS were added to each channel on the 10x chip. Cells were partitioned with Gel Beads into emulsion in the Chromium instrument where cell lysis and barcoded reverse transcription of RNA occurred following amplification. Single-cell RNAseq libraries were prepared by using the Chromium single cell 3′ library and gel bead kit v3 (10x Genomics, Pleasanton, CA). Sequencing was performed (as described above) and the digital expression matrix was generated by demultiplexing, barcode processing, and gene unique molecular index counting by using the Cell Ranger pipeline (10x Genomics, Pleasanton, CA). The data can be found under the GEO accession number: GSE156939

To identify different cell types and find signature genes for each cell type, the R package Seurat (version 3.1.2) was used to analyze the digital expression matrix. Cells with less than 500 unique molecular identifiers (UMIs) and greater than 50% mitochondrial expression were removed from further analysis. Seurat function NormalizeData was used to normalize the raw counts. Variable genes were identified using the FindVariableGenes function; genes with normalized expression values between 0.1 and 5 and with a dispersion of at least 0.5 were considered variable. The Seurat ScaleData function was used to scale and center expression values in the dataset for dimensional reduction. Principal component analysis (PCA), t-distributed stochastic neighbor embedding (t-SNE), and uniform manifold approximation and projection (UMAP) were used to reduce the dimensions of the data, and the first 2 dimensions were used in plots. A graph-based clustering approach was later used to cluster the cells; then signature genes were found and used to define cell types for each cluster. ECs were selected based on high expression of *PECAM1* and *CDH5* genes. SMCs were identified by the high expression of *ACTA2* and *TAGLN* genes. Module scores were calculated using the AddModuleScore function with default parameters.

*Western blots:*

Endothelial cells were lysed in modified RIPA buffer (50mM Tris pH 8, 0.1% SDS, 0.5% sodium desoxycholate, 1% triton-X100, 150mM NaCl, 1x protease inhibitor cocktail). Proteins were separated by SDS-PAGE gradient (4-20%) gel and transferred onto nitrocellulose membranes and incubated overnight at 4°C with primary antibodies. The following primary antibodies were used in this study: PLVAP (DSHB, Cat#MECA-32); PTGS1 (Cell Signaling Technologies, Cat#9896S); TXNIP (Cell Signaling, Cat#71632); FOXP1 (Cell Signaling Technologies Cat#4402S); TFRC (DSHB, Cat#G1/221/12); ZFP36L2 (Cell Signaling, Cat#2119); ACTN1 (Sigma, Cat#A5044); GAPDH (Millipore Sigma, Cat#MAB374); VCL (Millipore Sigma, Cat#V-9131). Secondary antibodies included: Amersham ECL Rabbit IgG HRP-Linked Whole Antibody (Cat#NA934) and Amersham ECL Mouse IgG, HRP-Linked Whole Antibody (Cat#NA931) both from Cytiva. Immuno-complexes were detected by enhanced chemiluminescence with SuperSignal™ West Pico PLUS (ThermoFisher Scientific #PI34580) and Femto Maximum Sensitivity Substrate (ThermoFisher Scientific #PI34096) using ChemiDoc™ Touch Imaging System (Bio-Rad Laboratories). Quantification of bands by densitometry analysis was performed using ImageLab Software (Bio-Rad Laboratories).

**Acknowledgments**: This work was supported by a grant from the National Institutes of Health R35HL140014 (M.L.I.A), Leducq Foundation (M.L.I.A. and M.V), R01HL147187 (C.E.R.), FAPESP 2016/19968-3 (V.F.), and K12 HD000849 (Y.A.), awarded to the Reproductive Scientist Development Program by the Eunice Kennedy Shriver National Institute of Child Health & Human Development, by the American College of Obstetricians and Gynecologists, as part of the Reproductive Scientist Development Program (Y.A.), and the Ruth L. Kirschstein National Research Service Award T32HL069766 (Y.A.). We thank Bill Brancart for his contributions to the Flow Profiler.

**References**

1. Iruela-Arispe ML, Davis GE. Cellular and molecular mechanisms of vascular lumen formation. Dev Cell. 2009;16(2):222-31.

2. Jackson DG. Leucocyte Trafficking via the Lymphatic Vasculature- Mechanisms and Consequences. Front Immunol. 2019;10:471.

3. Sun X, Feinberg MW. Regulation of endothelial cell metabolism: just go with the flow. Arterioscler Thromb Vasc Biol. 2015;35(1):13-5.

4. Vandenbroucke E, Mehta D, Minshall R, Malik AB. Regulation of endothelial junctional permeability. Ann N Y Acad Sci. 2008;1123:134-45.

5. Wettschureck N, Strilic B, Offermanns S. Passing the Vascular Barrier: Endothelial Signaling Processes Controlling Extravasation. Physiol Rev. 2019;99(3):1467-525.

6. Gomez-Salinero JM, Rafii S. Endothelial cell adaptation in regeneration. Science. 2018;362(6419):1116-7.

7. Apte RS, Chen DS, Ferrara N. VEGF in Signaling and Disease: Beyond Discovery and Development. Cell. 2019;176(6):1248-64.

8. Gerber HP, Dixit V, Ferrara N. Vascular endothelial growth factor induces expression of the antiapoptotic proteins Bcl-2 and A1 in vascular endothelial cells. J Biol Chem. 1998;273(21):13313-6.

9. Christensen PM, Liu CH, Swendeman SL, Obinata H, Qvortrup K, Nielsen LB, et al. Impaired endothelial barrier function in apolipoprotein M-deficient mice is dependent on sphingosine-1-phosphate receptor 1. FASEB J. 2016;30(6):2351-9.

10. Corada M, Orsenigo F, Bhat GP, Conze LL, Breviario F, Cunha SI, et al. Fine-Tuning of Sox17 and Canonical Wnt Coordinates the Permeability Properties of the Blood-Brain Barrier. Circ Res. 2019;124(4):511-25.

11. Trani M, Dejana E. New insights in the control of vascular permeability: vascular endothelial-cadherin and other players. Curr Opin Hematol. 2015;22(3):267-72.

12. Muller WA. Transendothelial migration: unifying principles from the endothelial perspective. Immunol Rev. 2016;273(1):61-75.

13. Wang S, Wang B, Shi Y, Moller T, Stegmeyer RI, Strilic B, et al. Mechanosensation by endothelial PIEZO1 is required for leukocyte diapedesis. Blood. 2022.

14. Choi JS, Seo TS. Orthogonal co-cultivation of smooth muscle cell and endothelial cell layers to construct in vivo-like vasculature. Biomicrofluidics. 2019;13(1):014115.

15. Cleuren ACA, van der Ent MA, Jiang H, Hunker KL, Yee A, Siemieniak DR, et al. The in vivo endothelial cell translatome is highly heterogeneous across vascular beds. Proc Natl Acad Sci U S A. 2019;116(47):23618-24.

16. Dayang EZ, Plantinga J, Ter Ellen B, van Meurs M, Molema G, Moser J. Identification of LPS-Activated Endothelial Subpopulations With Distinct Inflammatory Phenotypes and Regulatory Signaling Mechanisms. Front Immunol. 2019;10:1169.

17. Jambusaria A, Hong Z, Zhang L, Srivastava S, Jana A, Toth PT, et al. Endothelial heterogeneity across distinct vascular beds during homeostasis and inflammation. Elife. 2020;9.

18. Shathasivam P, Kollara A, Ringuette MJ, Virtanen C, Wrana JL, Brown TJ. Human ortholog of Drosophila Melted impedes SMAD2 release from TGF-beta receptor I to inhibit TGF-beta signaling. Proc Natl Acad Sci U S A. 2015;112(23):E3000-9.

19. Chiu JJ, Chien S. Effects of disturbed flow on vascular endothelium: pathophysiological basis and clinical perspectives. Physiol Rev. 2011;91(1):327-87.

20. Chien S. Mechanotransduction and endothelial cell homeostasis: the wisdom of the cell. Am J Physiol Heart Circ Physiol. 2007;292(3):H1209-24.

21. Nakajima H, Mochizuki N. Flow pattern-dependent endothelial cell responses through transcriptional regulation. Cell Cycle. 2017;16(20):1893-901.

22. Zhou J, Li YS, Chien S. Shear stress-initiated signaling and its regulation of endothelial function. Arterioscler Thromb Vasc Biol. 2014;34(10):2191-8.

23. Kim CW, Pokutta-Paskaleva A, Kumar S, Timmins LH, Morris AD, Kang DW, et al. Disturbed Flow Promotes Arterial Stiffening Through Thrombospondin-1. Circulation. 2017;136(13):1217-32.

24. Nakajima H, Yamamoto K, Agarwala S, Terai K, Fukui H, Fukuhara S, et al. Flow-Dependent Endothelial YAP Regulation Contributes to Vessel Maintenance. Dev Cell. 2017;40(6):523-36 e6.

25. Peng Z, Shu B, Zhang Y, Wang M. Endothelial Response to Pathophysiological Stress. Arterioscler Thromb Vasc Biol. 2019;39(11):e233-e43.

26. Polacheck WJ, Kutys ML, Yang J, Eyckmans J, Wu Y, Vasavada H, et al. A non-canonical Notch complex regulates adherens junctions and vascular barrier function. Nature. 2017;552(7684):258-62.

27. Ajami NE, Gupta S, Maurya MR, Nguyen P, Li JY, Shyy JY, et al. Systems biology analysis of longitudinal functional response of endothelial cells to shear stress. Proc Natl Acad Sci U S A. 2017;114(41):10990-5.

28. Chen BP, Li YS, Zhao Y, Chen KD, Li S, Lao J, et al. DNA microarray analysis of gene expression in endothelial cells in response to 24-h shear stress. Physiol Genomics. 2001;7(1):55-63.

29. Chu TJ, Peters DG. Serial analysis of the vascular endothelial transcriptome under static and shear stress conditions. Physiol Genomics. 2008;34(2):185-92.

30. Conway DE, Williams MR, Eskin SG, McIntire LV. Endothelial cell responses to atheroprone flow are driven by two separate flow components: low time-average shear stress and fluid flow reversal. Am J Physiol Heart Circ Physiol. 2010;298(2):H367-74.

31. Dekker RJ, van Soest S, Fontijn RD, Salamanca S, de Groot PG, VanBavel E, et al. Prolonged fluid shear stress induces a distinct set of endothelial cell genes, most specifically lung Kruppel-like factor (KLF2). Blood. 2002;100(5):1689-98.

32. Guo D, Chien S, Shyy JY. Regulation of endothelial cell cycle by laminar versus oscillatory flow: distinct modes of interactions of AMP-activated protein kinase and Akt pathways. Circ Res. 2007;100(4):564-71.

33. Burridge KA, Friedman MH. Environment and vascular bed origin influence differences in endothelial transcriptional profiles of coronary and iliac arteries. Am J Physiol Heart Circ Physiol. 2010;299(3):H837-46.

34. Lacorre DA, Baekkevold ES, Garrido I, Brandtzaeg P, Haraldsen G, Amalric F, et al. Plasticity of endothelial cells: rapid dedifferentiation of freshly isolated high endothelial venule endothelial cells outside the lymphoid tissue microenvironment. Blood. 2004;103(11):4164-72.

35. Sabbagh MF, Nathans J. A genome-wide view of the de-differentiation of central nervous system endothelial cells in culture. Elife. 2020;9.

36. Shima DT, Saunders KB, Gougos A, D'Amore PA. Alterations in gene expression associated with changes in the state of endothelial differentiation. Differentiation. 1995;58(3):217-26.

37. Brooks AR, Lelkes PI, Rubanyi GM. Gene expression profiling of vascular endothelial cells exposed to fluid mechanical forces: relevance for focal susceptibility to atherosclerosis. Endothelium. 2004;11(1):45-57.

38. Frueh J, Maimari N, Homma T, Bovens SM, Pedrigi RM, Towhidi L, et al. Systems biology of the functional and dysfunctional endothelium. Cardiovasc Res. 2013;99(2):334-41.

39. Maurya MR, Gupta S, Li JY, Ajami NE, Chen ZB, Shyy JY, et al. Longitudinal shear stress response in human endothelial cells to atheroprone and atheroprotective conditions. Proc Natl Acad Sci U S A. 2021;118(4).

40. Coon BG, Timalsina S, Astone M, Zhuang ZW, Fang J, Han J, et al. A mitochondrial contribution to anti-inflammatory shear stress signaling in vascular endothelial cells. J Cell Biol. 2022;221(7).

41. Baeyens N, Larrivee B, Ola R, Hayward-Piatkowskyi B, Dubrac A, Huang B, et al. Defective fluid shear stress mechanotransduction mediates hereditary hemorrhagic telangiectasia. J Cell Biol. 2016;214(7):807-16.

42. Mack JJ, Mosqueiro TS, Archer BJ, Jones WM, Sunshine H, Faas GC, et al. NOTCH1 is a mechanosensor in adult arteries. Nat Commun. 2017;8(1):1620.

43. Gallicchio M, Argyriou S, Ianches G, Filonzi EL, Zoellner H, Hamilton JA, et al. Stimulation of PAI-1 expression in endothelial cells by cultured vascular smooth muscle cells. Arterioscler Thromb. 1994;14(5):815-23.

44. Nackman GB, Bech FR, Fillinger MF, Wagner RJ, Cronenwett JL. Endothelial cells modulate smooth muscle cell morphology by inhibition of transforming growth factor-beta 1 activation. Surgery. 1996;120(2):418-25; discussion 25-6.

45. Powell RJ, Bhargava J, Basson MD, Sumpio BE. Coculture conditions alter endothelial modulation of TGF-beta 1 activation and smooth muscle growth morphology. Am J Physiol. 1998;274(2):H642-9.

46. Shirali AS, Romay MC, McDonald AI, Su T, Steel ME, Iruela-Arispe ML. A multi-step transcriptional cascade underlies vascular regeneration in vivo. Sci Rep. 2018;8(1):5430.

47. Boezio GL, Bensimon-Brito A, Piesker J, Guenther S, Helker CS, Stainier DY. Endothelial TGF-beta signaling instructs smooth muscle cell development in the cardiac outflow tract. Elife. 2020;9.

48. Birdsey GM, Dryden NH, Amsellem V, Gebhardt F, Sahnan K, Haskard DO, et al. Transcription factor Erg regulates angiogenesis and endothelial apoptosis through VE-cadherin. Blood. 2008;111(7):3498-506.

49. Laumonnier Y, Nadaud S, Agrapart M, Soubrier F. Characterization of an upstream enhancer region in the promoter of the human endothelial nitric-oxide synthase gene. J Biol Chem. 2000;275(52):40732-41.

50. Nikolova-Krstevski V, Yuan L, Le Bras A, Vijayaraj P, Kondo M, Gebauer I, et al. ERG is required for the differentiation of embryonic stem cells along the endothelial lineage. BMC Dev Biol. 2009;9:72.

51. Shah AV, Birdsey GM, Randi AM. Regulation of endothelial homeostasis, vascular development and angiogenesis by the transcription factor ERG. Vascul Pharmacol. 2016;86:3-13.

52. Yuan L, Nikolova-Krstevski V, Zhan Y, Kondo M, Bhasin M, Varghese L, et al. Antiinflammatory effects of the ETS factor ERG in endothelial cells are mediated through transcriptional repression of the interleukin-8 gene. Circ Res. 2009;104(9):1049-57.

53. Kalluri AS, Vellarikkal SK, Edelman ER, Nguyen L, Subramanian A, Ellinor PT, et al. Single-Cell Analysis of the Normal Mouse Aorta Reveals Functionally Distinct Endothelial Cell Populations. Circulation. 2019;140(2):147-63.

54. Boquest AC, Shahdadfar A, Fronsdal K, Sigurjonsson O, Tunheim SH, Collas P, et al. Isolation and transcription profiling of purified uncultured human stromal stem cells: alteration of gene expression after in vitro cell culture. Mol Biol Cell. 2005;16(3):1131-41.

55. Bork S, Pfister S, Witt H, Horn P, Korn B, Ho AD, et al. DNA methylation pattern changes upon long-term culture and aging of human mesenchymal stromal cells. Aging Cell. 2010;9(1):54-63.

56. Forsyth NR, Kay A, Hampson K, Downing A, Talbot R, McWhir J. Transcriptome alterations due to physiological normoxic (2% O2) culture of human embryonic stem cells. Regen Med. 2008;3(6):817-33.

57. Roobrouck VD, Vanuytsel K, Verfaillie CM. Concise review: culture mediated changes in fate and/or potency of stem cells. Stem Cells. 2011;29(4):583-9.

58. Crampton SP, Davis J, Hughes CC. Isolation of human umbilical vein endothelial cells (HUVEC). J Vis Exp. 2007(3):183.

59. Langmead B, Salzberg SL. Fast gapped-read alignment with Bowtie 2. Nat Methods. 2012;9(4):357-9.

60. Dobin A, Davis CA, Schlesinger F, Drenkow J, Zaleski C, Jha S, et al. STAR: ultrafast universal RNA-seq aligner. Bioinformatics. 2013;29(1):15-21.

61. Szklarczyk D, Franceschini A, Wyder S, Forslund K, Heller D, Huerta-Cepas J, et al. STRING v10: protein-protein interaction networks, integrated over the tree of life. Nucleic Acids Res. 2015;43(Database issue):D447-52.

62. Zhou Y, Zhou B, Pache L, Chang M, Khodabakhshi AH, Tanaseichuk O, et al. Metascape provides a biologist-oriented resource for the analysis of systems-level datasets. Nat Commun. 2019;10(1):1523.

63. Wohlschlegel JA. Identification of SUMO-conjugated proteins and their SUMO attachment sites using proteomic mass spectrometry. Methods Mol Biol. 2009;497:33-49.

64. Florens L, Carozza MJ, Swanson SK, Fournier M, Coleman MK, Workman JL, et al. Analyzing chromatin remodeling complexes using shotgun proteomics and normalized spectral abundance factors. Methods. 2006;40(4):303-11.

65. Perez-Riverol Y, Csordas A, Bai J, Bernal-Llinares M, Hewapathirana S, Kundu DJ, et al. The PRIDE database and related tools and resources in 2019: improving support for quantification data. Nucleic Acids Res. 2019;47(D1):D442-D50.

66. Kim S, Pevzner PA. MS-GF+ makes progress towards a universal database search tool for proteomics. Nat Commun. 2014;5:5277.

67. Serang O, MacCoss MJ, Noble WS. Efficient marginalization to compute protein posterior probabilities from shotgun mass spectrometry data. J Proteome Res. 2010;9(10):5346-57.

68. Granholm V, Kim S, Navarro JC, Sjolund E, Smith RD, Kall L. Fast and accurate database searches with MS-GF+Percolator. J Proteome Res. 2014;13(2):890-7.

69. The M, MacCoss MJ, Noble WS, Kall L. Fast and Accurate Protein False Discovery Rates on Large-Scale Proteomics Data Sets with Percolator 3.0. J Am Soc Mass Spectrom. 2016;27(11):1719-27.

70. MacLean B, Tomazela DM, Shulman N, Chambers M, Finney GL, Frewen B, et al. Skyline: an open source document editor for creating and analyzing targeted proteomics experiments. Bioinformatics. 2010;26(7):966-8.

71. Choi M, Chang CY, Clough T, Broudy D, Killeen T, MacLean B, et al. MSstats: an R package for statistical analysis of quantitative mass spectrometry-based proteomic experiments. Bioinformatics. 2014;30(17):2524-6.

72. Dardik A, Chen L, Frattini J, Asada H, Aziz F, Kudo FA, et al. Differential effects of orbital and laminar shear stress on endothelial cells. J Vasc Surg. 2005;41(5):869-80.

**Figure 1.** **Human umbilical cord endothelial cell transcriptome.**

**A.** Model of endothelial cell collection *for in vivo* (cord) and *in vitro* (culture) experiments. Endothelial cells are isolated in a slurry and used immediately for downstream experiments or cultured for subsequent passages. **B.** Principal component analysis (PCA) of transcriptome of the 7 matched cord, early culture and late culture samples with significant separation along PC1. **C.** Spearman-correlation demonstrating inter-condition (cord=C, early passage-=E, late passage=L) and intra sample variability with k-means clustering by cord **D.** 40-45% of the of the expressed genes overlapped in relative expression patterns between cord and culture regardless of early and late culture. While early and late culture overlap in 93% of the genes. **E.** Volcano plot of genes most significantly expressed in cord (right) versus culture (left) by log10 fold change. **F.** Heatmap of top 30 differentially expressed genes in 21 samples from 7 individuals expressed between cord and culture **G.** Network profile of subset of Gene Expression Omnibus (GEOs) significant in cord versus culture. GEO is represented by cluster identity and each term is represented as circle node visualized on Metascape. The highlighted GEOs are the most significant pathways by p-value **H.** Mass spectrometry proteomic profile of 7 matched cord and culture separated by cord and culture on PC1. **I.** Scatter plot depicting RNA t-statistics (cord/culture) versus protein t-statistics (cord/culture) with a correlation coefficient of r=0.4.

**Figure 2.** **Shear stress induces a time-dependent transcriptomic flow signature**

**A.** Phenotype of *in vitro* flow model induces endothelial cellular shape changes under flow. **B.** PCA of each sample under static (yellow) and flow-conditioned endothelial cells by bulk RNA-seq (blue) **C.** Heatmap of differentially expressed genes (DEGs) by bulk RNA-seq in static and flow-conditioned cells. Row z-score reflects the gene expression change. **D.** Volcano plot illustrating statistical significance versus fold change between flow and static cultures demonstrating the most significantly differentially expressed genes. **E.** Time-dependent volcano plot and correlation coefficient highlighting the correlation of flow time to cord transcriptome where longer flow correlates more strongly to cord (in vivo) transcriptome. **F.** Network profile of subset of GEOs significant in flow versus static culture. GEO is represented by cluster identity and each term is represented as circle node visualized with Metascape.

**Figure 3.** **Flow rescues a degree of the cord transcriptome**

**A.** PCA demonstrates stark differences in cord (*in vivo*) versus culture (*in* vitro) along PC1. Flow rescues the transcript from the culture towards the cord along PC1. Based on the transcriptional similarity of the different flow time points (Figure 2B) we consolidated the “under flow” label for Figure 3A for clarity. Based on the sample distribution on PC1 (from left to right: cord > extended flow > early culture + short-term flow > culture) and its magnitude (31% of the covariance in the dataset). PC1 primarily represents the differences between Cord and Culture samples (we interpret the PC2 to represent differences between short-term and extended flow). The middle position between cord and late culture, is a partial rescue of the differences imparted by culture. **B.** Venn-diagram demonstrating the significant number of differentially expressed genes by condition and its concordant or discordant correlation to each another **C.** Correlation heatmap of top 10 module eigengenes (ME) by experimental condition, cord versus culture or flow versus static. The columns are labeled by experimental condition. The rows are labeled by the ME color. The biweight midcorrelation coefficients are shown numerically for each cell, with the significance of the correlation shown immediately below (FDR). Cells are colored based on the strength and sign of the correlation. **D.** Cluster dendrogram and module assignment for RNA modules from weighted gene co-expression network analysis (WGCNA). Identification of gene co-expression modules using average hierarchical linkage clustering. The vertical axis denotes the co-expression distance, and the horizontal axis corresponds to genes. Dynamic tree cutting was applied to identify modules by dividing the dendrogram at significant branch points. Modules are displayed with different colors in the horizontal bar immediately below the dendrogram, with gray representing unassigned genes. Correlation coefficients with experimental conditions are also represented based on strength and direction (negative correlations to positive correlations ranging from blue to red). **E-F.** Eigengene value of flow-dependent rescue of the blue module; C=cord, E=early, L=late and enriched blue-module GEO. **G-H.** Eigengene value of flow-dependent rescue of the brown module and enriched GEO.

**Figure 4.** **Protein validation of transcripts affected by flow**

**A**. Western blot analysis of three examples of transcripts that are regulated by flow in a concordant manner (up regulated, downregulated and unchanged) in relation to cord isolated lysates. Uncropped data is shown in figure 4-source data 1. **B**. Quantification of the same genes using three independent biological replicates. Each color represents an independent experiment. Numbers show p value of ANOVA analysis between the time points.

**Figure 5.** **Endothelial cell-smooth muscle cell interactions**

**A.** Schematic overview of single-cell RNAseq experiments. **B.** UMAP of scRNAseq data with four distinct clusters with 2 technical replicates (labeled A/B) as indicated in the legend. **C.** Identity of endothelial cells was confirmed by expression of CDH5 and PECAM transcripts**. D.** UMAP of scRNAseq for endothelial cell mono-culture (EC-MC) and endothelial cell co-culture (EC-CC) with biological replicates as indicated. **E.** Identity of endothelial cells was confirmed by expression of CDH5 and PECAM transcripts**. F.** Heatmap identifying the top differentially expressed genes with log fold>2 for each condition relative to the other cell types. **G.** Ingenuity analysis demonstrates most significantly upregulated module score based on growth factors, cytokines, and transcription factors. **G.** TGFB1 and VEGF show the highest module score in co-culture relative to endothelial cell monoculture **I.** TGFB1 activated genes are upregulated in co-culture **J.** Clathrin family members are upregulated in co-culture; whereas caveolin family members are decreased in co-culture **K.** Endothelial cell makers are unchanged and stable in mono- and co-culture endothelial cells

**Figure 6.** **Co-cultured endothelial cells with smooth muscle cells rescue a cohort of genes when compared to the cord transcriptome**

**A.** UMAP of endothelial cell co-culture (EC-CC) with smooth muscle cells versus endothelial cell-monoculture (EC-MC) in relation to endothelial cells isolated directly from umbilical cord. Insert: confirmed endothelial cell identity by PECAM. **B.** Ingenuity analysis demonstrates most significantly upregulated module score based on growth factors, cytokines, and transcription factors. **C.** PDGFB, the most significantly upregulated growth factor, is rescued by co-culture. **D.** Environment dependent transcriptional enrichment demonstrated by UMAP. **E.** Dotplot illustrates the top markers of in cord, monoculture (MC), and co-culture (CC). Dot size corresponds to the proportion of cells within the group expressing each transcript and dot color intensity corresponds to the expression level. **F.** Violin plot of environment-dependent (heterotypic co-culture) gene expression illustrating examples of genes rescued (AKK1, EPN2, TJP1, TMEM16S, TMEM203) and non-rescued genes (KLF2, KLF4, ELN, ADGRA2, TEK).

**Figure 7. Summary Figure.** Schematic representing experimental design, culture conditions, and corresponding validated genes changes.

**Figure 1-figure supplement 1. A**. Left. Hematoxylin and eosin (H&E) stain of an axial section of an umbilical cord depicting two umbilical arteries and a large umbilical vein (*). Right. En-face staining of endothelial cells of the umbilical vein with DAPI and CHD5. **B.** Middle. H&E of umbilical cord with endothelial cells (EC) lining the inner surface and smooth muscle cells (SMC) below. Staining for CDH5 in EC and smooth muscle actin (SMA) in SMCs both in red. **C.** tSNE plot from 3 participants (A, B, C) demonstrating 10 clusters (0-9) nearly all PECAM1 positive, PTPRC/CD45 negative, with rare red blood cells RBCs  **D.** Phase-contrast images of cells at different passage (p) 2 and 7. **E.** Flow cytometry demonstrates isolated cord cells are PECAM+ **F.** Immunofluorescence staining at 40x under static and flow conditions for the same participants stained for CDH5, Tubulin, and DAPI.

**Figure 1-figure supplement 2. A**. Assessment of inter- and intra- participant variability with transcriptomic correlation of coefficient between and within culture treatment. **B.** Network analysis for cord vs culture depicted as protein connectivity. Each node network is assigned a unique color. GO enrichment analysis was applied to each network to assign “meanings” to the network component. **C.** PCA of cord versus early culture with 29.8% variance along PC1**. D.** Volcano plot of statistical significance against fold change between cord and static culture (early and late) as well as early vs late culture demonstrating the most significantly differentially expressed genes. **E.** Venn-diagram of protein change seen by LC-MS in cord versus culture and overlap. **F.** Volcano plot of protein signature seen in cord versus cultured endothelial cells where significance is demonstrated in red with select proteins highlighted. **G.** Heatmap correlating the t-statistics for protein versus RNA changes in cord versus culture in dataset. Red is up regulated protein or gene whereas blue is downregulated.

**Figure 2-figure supplement 1. A.** Venn diagram depicting differentially expressed gene number by condition and directionality of change. **B-C.** Gene set enrichment analysis of differential genes in cultured endothelial cells under flow (versus static) revealed gene annotations related to an acute inflammatory response, heart morphogenesis, second messenger-mediated signaling, and ossifications. We also found tRNA and rRNA metabolic processes were attenuated under flow. **D.** Heatmap compares well-known flow-responsive genes (NOS3, VCAM1, ICAM1, KLF2, KLF4) identified by this dataset. There is a clear increase in known flow responsive genes, consistent with what has been shown in the literature. **E.** Volcano plot of flow response genes over time (0.5hr, 8hr, 24hr, and 48hr) between static culture and flow demonstrating the most significantly differentially expressed genes.

**Figure 2-figure supplement 2.** Flow profiler application files.

**Figure 2-figure supplement 3.** Operation manual and explanatory information for the Flow Profiler.

**Figure 3-figure supplement 1. A-C.** Cluster dendrogram and module assignment for RNA modules from weighted gene co-expression network analysis (WGCNA). Identification of gene co-expression modules using average hierarchical linkage clustering. The vertical axis denotes the co-expression distance, and the horizontal axis corresponds to genes. Dynamic tree cutting is applied to identify modules by dividing the dendrogram at significant branch points. Modules are displayed with different colors in the horizontal bar immediately below the dendrogram, with gray representing unassigned genes. Correlation coefficients with experimental conditions are also represented based on strength and direction (negative correlations to positive correlations ranging from blue to red). **A.** is specific for cord, culture and flow. **B.** by time under flow and **C.** is by participant level. **D.** Eigengene value of flow-dependent rescue of the blue module; C=cord, E=early, L=late and enriched blue-module GEO. **E.** Eigengene value of condition and flow-dependent rescue of the blue module.

**Figure 5-figure supplement 1. A-B.** Graphical description of experimental design of scRNAseq co-culture experiment and violin plot of quality metrics of RNA, counts, and mitochondrial genes. Two participants combined to define “culture” of endothelial cell monolayer. Two different participant smooth muscle cells (SMC) and a co-culture. For co-culture experiments primary umbilical smooth muscle cells were seeded sub-confluent onto the culture plate 24h prior to seeding HUVECs at confluent density. After an additional 24h co-cultured cells were trypsinized. Dot plot demonstrates the top markers of in cord, monoculture (MC), and co-culture (CC). **C.** UMAP by cluster and sample demonstrating EC (PECAM1) and SMC (TAGL1) population. **D.** Dot plot demonstrating purity of experimental design by UMAP cluster. **E.** Endothelial cell monolayer (MC) and co-culture (CC) scRNAseq analysis demonstrates differentially expressed gene expression. Dot size corresponds to the proportion of cells within the group expressing each transcript and dot color intensity corresponds to the expression level.

**Figure 4-source data 1.** Source file depicting uncropped Western blot analysis from Figure 4 (Novel genes affected by flow). On the left are six blots with the protein of interest and molecular weight of anticipated protein on the Y-axis with both colorimetric overlay and chemiluminescence image. The protein of interest is squared in red. If the blot had an additional 2^nd^ or 3^rd^ antibody probe it is depicted serially in a column with the protein of interest and molecular weight as marked.

**Supplementary File 1**. RNAseq gene expression matrix by participant and environmental level conditions (cord, early culture, and late culture)

**Supplementary File 2**. LC-MS protein expression by participant and environmental level conditions (cord, culture).

**Supplementary File 3**. RNAseq gene comparison matrix by environmental condition (multi-tabulated excel): Tabs included (n=10) are Cord vs Early, Cord vs Late, Cord vs Early/Late, Early vs Late, Culture vs 0.5hr flow, Culture vs 8hr flow, Culture vs 24hr flow, Culture vs 48hr flow, Flow (>8hr) vs No Flow, Early flow vs No flow

**Supplementary File 4**. Biweight midcorrelation (bicor) between gene expression levels by condition and participant with module correlation (multi-tabulated excel): Tabs included (n=2): all genes, non-coding genes

**Supplementary File 5**. Environmental driven gene expression and rescue with concordance and discordance (multi-tabulated excel): Table included (n=6): cord, flow, up in both, down in both, up in cord, down in flow, down in cord, up in flow.

**Supplementary File 6**. scRNAseq gene matrix of endothelial cell monolayer versus co-culture (MC vs CC)

**Supplementary File 7**. scRNAseq gene matrix of endothelial cell monolayer (MC) versus cord.

**Supplementary File 8**. scRNAseq gene matrix of endothelial cell – smooth muscle cell co-culture (CC) versus cord.

**Supplementary File 9**. Concordance of differentially expressed RNA and protein in cord versus culture.
